# Joint clinical determinants for bivariate hematological parameter among TB/HIV co-infected adults under TB/HIV treatment in university of Gondar comprehensive specialized hospital: Retrospective panel data study

**DOI:** 10.1186/s13104-024-06808-6

**Published:** 2024-06-01

**Authors:** Nurye Seid Muhie

**Affiliations:** Department of Statistics, Mekdela Amba University, Tulu Awulia, Ethiopia

**Keywords:** TB/HIV co-infected, Common predictors, Hematological parameter, Hematocrit, Hemoglobin, Longitudinal sub model

## Abstract

**Background:**

Worldwide ranking above HIV/AIDS, tuberculosis is continues to have a significant effect on public health and the leading cause of death due to high progression of HIV. The objective of current study was identify joint clinical determinants that affecting bivariate hematological parameter among TB/HIV co-infected adults under TB/HIV treatment in university of Gondar comprehensive specialized hospital.

**Method:**

The result of these study was conducted at university of Gondar comprehensive specialized hospital, Gondar, Ethiopia by using a retrospective cohort follow up study from September 2015-march 2022 G.C. The source of data in this study was secondary data obtained from patients chart. Bayesian approach of longitudinal linear mixed effect sub model was used in panel data set to get wide range of information about TB/HIV co-infected patients.

**Result:**

Out of 148 co-infected participants more than half of the patients (56.1%) and (52.7%) accounted for CPT and INH non users, of which 10.8% and 10.3% had the outcome of mortality respectively. The random intercept and slope model were selected for repeated measure hemoglobin level and hematocrit based on deviance information criteria (DIC), and probability of direction (Pd) under the full model.

**Conclusion:**

Current study revealed that clinical predictors red blood cell count, platelet cell count, fair and good treatment adherence, other ART regiment, IPT drug users, and viral load count < 10,000 copies/mL, were associated with high hemoglobin level concentration while, lymphocyte count, WHO clinical stage-IV,1e ART regiment, and patients with OIs results for low hemoglobin level concentration. Likewise, red blood cell count, platelet cell count, fair and good treatment adherence, IPT drug users, and viral load count < 10,000 copies/mL co-infected patients had high hematocrit, while lymphocyte count, WHO clinical stage-III,1c ART regiment, and patients with OIs significantly leads to low hematocrit. Health professionals give more attention to these important predictors to reduce progression of disease when the co-infected patients come back again in the hospital. In addition, health staff should conduct health related education for individuals to examine continuous check-up of co-infected patients.

## Introduction

Human immunodeficiency virus (HIV) pandemic presents a massive challenge to the control of tuberculosis (TB) at all levels [1].HIV infection is worsening the co-infected of TB [2] and TB is the major clinical indication of patients with high progression of HIV [3]. Tuberculosis is continues to have a significant effect on public health [4] and the leading cause of death from a single infectious agent, ranking above HIV/AIDS worldwide. Ten million individuals affected with tuberculosis worldwide in 2021, making this TB was the most common infectious disease and one of the top ten causes of death [1, 3]. In the same year 2 million deaths due to tuberculosis occur throughout the world [5] and half of the people affected by tuberculosis (TB) worldwide are found in sub-Saharan Africa [6–8].

The interaction between HIV and TB epidemics had suspiciously affects people in Africa [9]. In high HIV prevalence population, TB is a leading cause of morbidity and mortality, and HIV is driving the TB epidemic in many countries, especially those in sub-Saharan Africa [1]. Those indicated sub-Saharan Africa is facing the worst tuberculosis epidemic [10–12] due to high progression of HIV epidemic [13, 14].

According to world health organization (WHO) evaluation lists Ethiopia is one of the 20 countries with the highest burden of HIV/TB co-infection worldwide [15]. HIV/AIDS is among the top ten high burden counties with an incidence rate of 341/100,000 of which 31% of TB patients are living with HIV [16, 17]. These indicated, TB/HIV co-infection is the leading cause of death in people living with HIV/AIDS [18]. The number of Ethiopians receiving antiretroviral medication (ART) for co-infection with HIV and tuberculosis is rising annually. However, In Ethiopia, co-infection with HIV and TB results in a variety of problems, such as difficulties with diagnosis and treatment follow-up in medical settings and even death [17, 19]– [21].Hematological parameter of co-infected patients such as hemoglobin level and hematocrit concentration decreased [22–25].

TB/HIV co-infected patients studies have been done in different previous literature. Some of them have been conducted to determine the predictors for hemoglobin level and hematocrit separately [22–30], which is not done jointly for better understanding the effect of these hematological biomarkers. In order to gain a better understanding of the modeling of co-infection of TB/HIV and the various factors influencing the hematological parameter of this patient population in the region of Ethiopia, particularly in the Amhara region, increased TB spread is still a contentious issue and a top priority goal.

So, the former literature shows as some mentioned above, a scarce of study on TB/HIV co-infected patient’s hemoglobin level and hematocrit outcomes jointly and related predictors in university of Gondar comprehensive specialized hospital, Ethiopia. Moreover, this is the first study to identify the effect of clinical predictors on TB/HIV co-infected patients hemoglobin level and hematocrit jointly. The present study was aimed to identify the joint clinical determinants that affecting bivariate hematological parameter (hemoglobin level and hematocrit) among TB/HIV co-infected adults under TB/HIV treatment in university of Gondar comprehensive specialized hospital. The findings from this study will contribute to the body of knowledge that informs TB-HIV program planers, decision makers, and project implementers by providing joint clinical predictors of hemoglobin level and hematocrit among TB-HIV co-infected patients during ART/TB treatment.

## Materials and methods

### Study area

This study was conducted at university of Gondar comprehensive specialized hospital.

### Study design

In these TB/HIV co-infected patients, retrospective cohort follow up study was performed to retrieve necessary information.

### Study population

Adult TB/HIV co-infected patients were considered as the study population.

### Study period

Adult TB/HIV co-infected patients whose treated treatment between September 2015-March 2022 G.C.

### Source of data

The secondary data sources were considered in this study obtained from patient’s chart.

### Inclusion criteria

This study was considered adult TB/HIV co-infected patients who had at least two visit times (one year) for bivariate hematological parameter (hemoglobin and hematocrit), patients whose age ≥ 15 years, and started TB/HIV treatment within follow-up study period (September 2015- March 2022 G.C).

### Exclusion criteria

This study was doesn’t considered about adult TB/HIV co-infected patients who had only one visit times (six months) for hemoglobin and hematocrit, patients whose age < 15 years, and started TB/HIV treatment without follow-up study period.

### Baseline sample size determination

Based on inclusion and exclusion criteria, the final baseline sample size for this study was 148 participants.

### Data collection procedure

In these study medical registration number (MRN) can be used for selection of co-infected patient charts. Then, from the review of co-infected patient charts the necessary information were retrieved by two trained TB/HIV data collectors. The data collectors spent three months for extracting the data.

### Data collection quality

In order to ensure the quality of collected data one day intensive training was given to data collectors. Before the actual data collected, the adequacy of checklist was evaluated and ambiguous questions are modified. The necessary amendments are made on the final data extraction format for completeness and consistency and the full formats are checked by TB/HIV data management.

### Variables included in the study

#### Response variable

The response variable in this study was bivariate hematological parameter (hemoglobin level per deciliter and hematocrit in percent) among TB/HIV co-infected patients.

#### Independent variable

Clinical independent variables for TB/HIV co-infected patients in this study were white blood cell (WBC) in 10^^3^/$\mu l$, red blood cell (RBC) in 10^^6^/$\mu l$, Platelet cell count in 10^^3^/$\mu l$, lymphocyte count in %, monocyte count, CD4 cell count, viral load count, treatment adherence, other comorbid condition (OCC), Cotrimoxazole preventive therapy (CPT), Isoniazid preventive therapy (IPT), antiretroviral (ART) Regiment, opportunistic infection (OIs) other than tuberculosis, WHO clinical stage, and tuberculosis type.

### Method of data analysis

The data was collected by Microsoft excel and imported in to SPSS version 26 and finally the data imported in to R-software version 4.1.3 for statistical analysis by using 5% level of significance.

### Exploratory data analysis

Data exploration was used to investigate various associations, structures, patterns and gives highlight about the nature of the data. In this study the individual profile plot with mean structure can be considered in the data set in order to gain some visions of the data related with hemoglobin level and hematocrit.

### Longitudinal sub-model

The longitudinal data is actually collected with error at time points ${t}_{ij}$ for each subject. To achieve this we postulate a suitable intermittently Bayesian linear mixed model to describe the subject-specific time evolutions for each response. The observed longitudinal responses ${(y}_{kij},k=1)$ are measured with an error. To minimize these errors of the data, we can utilized the Bayesian linear mixed model under longitudinal sub model by estimating the true underlying, and complete, subject-specific trajectory function (${ m}_{i}\left(t\right))$. Hence, longitudinal sub model can be formulated as:

$${y}_{ki}\left(t\right)={{X}^{{\prime }}}_{ki}\left(t\right){\beta }_{k}+{{Z}^{{\prime }}}_{ki}\left(t\right){b}_{ki}+{\epsilon }_{ki}\left(t\right)={m}_{ki}\left(t\right){+\epsilon }_{ki}\left(t\right)$$

Where $${\beta }_{k}$$ is the corresponding vector of the fixed effects, $${X{\prime }}_{ki}\left(t\right)$$ is the design matrix of fixed effects, including time effects and baseline covariates, $$b{\rm{\_}}ki \sim N{\rm{(0,}}\Psi {\rm{ )}}$$ is the corresponding vector of random effects ,$${Z{\prime }}_{ki}\left(t\right)$$ is the design matrix of (size n $$\times$$q) random effects covariates, $${\epsilon }_{ki}\left(t\right)$$is the corresponding measurement error term and distributed as $${\epsilon }_{ki}\left(t\right)\sim N(0,{\delta }_{K}^{2}{I}_{ni}$$) and $${ m}_{ki}\left(t\right)=$$$${X}_{ki}\left(t\right)\beta +{Z}_{ki}\left(t\right){b}_{ki}$$ is the trajectory function.

### Model selection criteria

To select the better random effect model, the BIC and pD are the most commonly used methods of model selection criteria for Bayesian linear mixed model. Therefore, the model with the smallest value of BIC and pD among Bayesian linear mixed model is the appropriate model [31].

## Results

### Clinical characteristics of adult TB/HIV co-infected patients

Out of 148 co-infected participants less than half (47.3%) were CD4 cell count less than 200cells/mm^3^, of which 40.0% death due to TB/HIV. Similarly, less than one third of participants (31.1%) were viral load count less than 10,000 copies/mL, of which 39.1% had mortality from the disease. Likewise, less than one fourth of co-infected patients (24.3%) were good treatment adherence, of which 11.1% had mortality from the disease. Around more than half of the patients 56.1% and 52.7% of the study participants had not CPT and IPT users, of which 10.8% and 10.3% had the outcome of mortality respectively. Considering ART Regiment, around 15% had 1e ART treatment and 32% leads to mortality form the disease. More than half of WHO clinical stage-IV had 56.5% death form TB/HIV. Considering types of TB, around more than half of the patients (57.4%) were Extra-Pulmonary TB, of which (27.1%) leads to death from disease. The minimum values of WBC in 10^^3^/$\mu l$(2.60), RBC in 10^^6^/$\mu l$(2.0), platelet cell count in 10^^3^/$\mu l$(20), lymphocyte count in %(21.0), and monocyte count in % (2.3) and the maximum value of WBC in 10^^3^/$\mu l$ (10.9), RBC in 10^^6^/$\mu l$ (8.1), platelet cell count in 10^^3^/$\mu l$(566), lymphocyte count in %(69.1), and monocyte count in %(55.4) respectively (Table 1).

### Repeated measure hemoglobin level and hematocrit exploratory data analysis

**Fig. 1:**
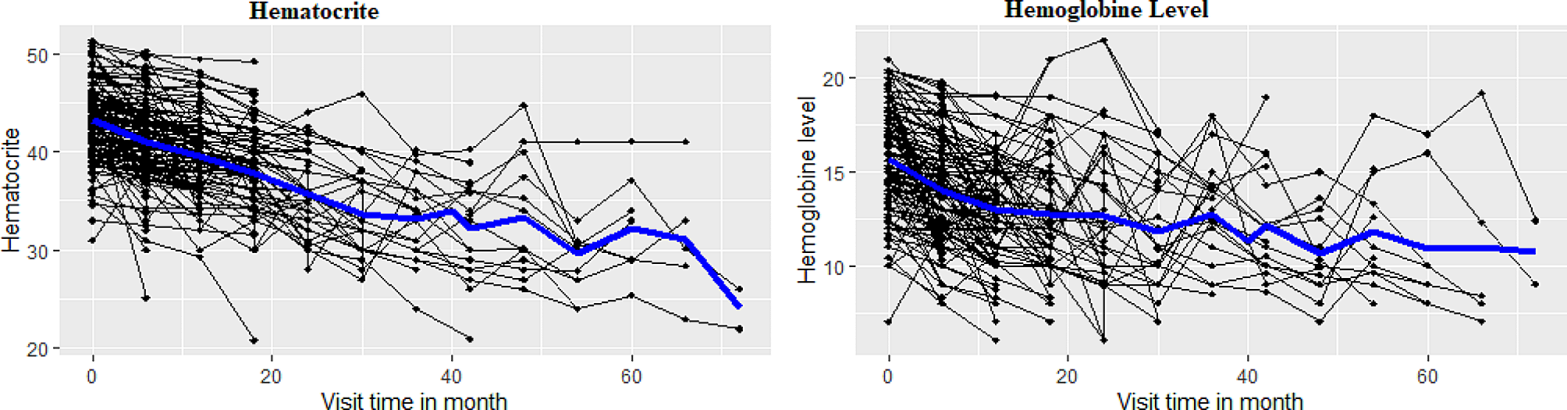
Smooth profile plot and mean structure for hemoglobin level and hematocrit

Smooth profiles plot with the mean structure was used to determine the general trend over time and provide information about the change at given times. Hemoglobin level and hematocrit for TB/HIV co-infected patients fluctuates through visit time. Then, blue line indicated visit time of co-infected patient’s enlarged the average hemoglobin level and hematocrit was showed to be decrement variation (Fig. 1). Co-infected patients decrement variation in hemoglobin level and hematocrit results for high chance of death.

**Table 1:** Baseline Clinical Characteristics of TB/HIV co-infected patients

Variables	Categories	Death (%)	Total (%)	
CD4 cell count	< 200cells/mm^3^	28(40.0)	70(47.3)	
	>=200cells/mm^3^	11(14.1)	78(52.7)	
Viral load count	>=10,000	21(20.6)	102(68.9)	
	< 10,000	18(39.1)	46(31.1)	
Adherence	Poor	15(30.6)	49(33.1)	
	Fair	20(31.7)	63(42.6)	
	Good	4(11.1)	36(24.3)	
OIs	No	12(11.3)	106(71.6)	
	Yes	27(64.3)	42(28.4)	
OCC	No	17(14.9)	114(77.0)	
	Yes	22(64.7)	34(23.0)	
CPT	No	9(10.8)	83(56.1)	
	Yes	30(46.2)	65(43.9)	
IPT	No	8(10.3)	78(52.7)	
	Yes	31(44.3)	70(47.3)	
ART Regiment	1d	15(33.3)	45(30.4)	
	1c	10(20.4)	49(33.1)	
	1e	7(31.8)	22(14.9)	
	Others	7(21.9)	32(21.6)	
WHO Clinical Stage	Stage-I	7(14.9)	47(31.8)	
	Stage-II	5(12.2)	41(27.7)	
	Stage-III	14(37.8)	37(25.0)	
	Stage-IV	13(56.5)	23(15.5)	
Types of TB	Pulmonary	16(25.4)	63(42.6)	
	Extra-Pulmonary	23(27.1)	85(57.4)	
**Continuous Variables**	**Minimum**	**Maximum**	**Mean**	**Standard deviation**
WBC in 10^^3^/$\mu l$	2.60	10.90	5.9074	1.70816
RBC in 10^^6^/$\mu l$	2.00	8.10	4.0813	1.12154
Platelet in 10^^3^/$\mu l$	20	566	265.09	99.187
Lymphocyte in %	21.0	69.1	45.196	12.3547
Monocyte in %	2.3	55.4	6.603	12.3547

### Random effect model selection

In this study based on deviance information criteria (DIC), and probability of direction (Pd) under the full model, the random intercept and slope model (model-IV) were selected for repeated measure hemoglobin level and hematocrit (Table 2).

**Table 2:** Random effect model selection for repeated measure hemoglobin level and hematocrit

Model	Random effect model		Null model		Full model	
	Intercept only model	Intercept and slope model	DIC	pD	DIC	pD
I.	Hemoglobin	-	6477.281	366.8369	6483.718	830.0254
	Hematocrit	-				
III.	Hematocrit	-	6461.998	559.51	6506.393	671.1561
	-	Hemoglobin				
V.	Hemoglobin	-	6512.13	550.7604	6498.982	628.9727
	-	Hematocrit				
VII.	-	Hemoglobin	6605.337	848.1201	6433.964	384.8114
	-	Hematocrit				

From bivariate Bayesian linear mixed sub model analysis showed that the covariates visit time in month, red blood cell (RBC), platelet cell count, lymphocyte count, treatment adherence, WHO clinical stage, ART regiment, OIs, OCC, IPT drug users and viral load count were significant clinical predictors affected hemoglobin level at 5% level of significance (Table 3). Likewise, at 5% level of significance the clinical predictors affected hematocrit of TB/HIV co-infected patients were visit time in month, red blood cell (RBC), platelet cell count, lymphocyte count, monocyte count, treatment adherence, WHO clinical stage, ART regiment, OIs, viral load count, IPT and CPT drug users (Table 4).

Therefore, from the analysis of bivariate Bayesian linear mixed sub-model, the joint clinical predictors that affected bivariate hematological parameter (hemoglobin level and hematocrit) jointly were red blood cell (RBC), platelet cell count, lymphocyte count, treatment adherence, WHO clinical stage, ART regiment, OIs, IPT drug users, and viral load count (Tables 3 and 4).

**Table 3:** Bivariate Bayesian linear mixed model result for hemoglobin level

Variables	Categories	Values	Standard error	*p*-values	95% CI	
					Lower	Upper
Intercept	-	16.0280	0.0570	0.000*	13.4992	18.7164
Visit time	-	-0.1773	0.0005	0.000*	-0.2116	-0.1428
WBC	-	-0.0851	0.0037	0.478	-0.3104	0.1409
RBC	-	0.1365	0.0071	0.016*	0.5477	1.2756
Platelet cell count	-	0.0000	0.0019	0.0001*	0.0036	0.0040
Lymphocyte	-	-0.0225	0.0006	0.0165*	-0.0541	-0. 0116
Monocyte	-	-0.0690	0.0015	0.132	-0.1606	0.0182
Adherence (poor)	Fair	0.6118	0.0166	0.024*	0.0740	1.4056
	Good	1.1726	0.0190	0.048*	1.0160	2.2996
WHO(Ref = Stage-I)	Stage-II	1.8059	0.0183	0.103	-0.2904	1.8834
	Stage-III	0.6176	0.0178	0.280	-0.4758	1.7111
	Stage-IV	-0.2695	0.0217	0.006*	-1.4683	-0.0217
ART regiment (Ref = 1d)	1c	0.0859	0.0180	0.880	-0.9551	1.1397
	1e	1.2574	0.0208	0.010*	0.2918	2.0930
	Other	1.0358	0.0191	0.030*	1.0142	2.2088
OIs(Ref = No)	Yes	-0.2734	0.0201	0.016*	-1.4240	-0.0823
OCC(Ref = No)	Yes	-0.1882	0.0214	0.045*	-1.1481	-0.5720
CD4cell(Ref = < 200cells/mm^3^)	>=200cells/mm^3^	0.2588	0.0142	0.578	-0.6054	1.1766
IPT(Ref = No)	Yes	1.0404	0.0167	0.020*	1.0052	2.9102
CPT(Ref = No)	Yes	-0.0028	0.0212	0.988	-1.2531	1.3752
Viral load (Ref=>=10,000)	< 10,000	0.5792	0.0161	0.021*	0.5208	2.2873
Types of TB (Ref = pulmonary)	Extra-pulmonary	-0.0666	0.0234	0.040*	-1.5470	-0.0393
Sigma	-	1.9787	0.0022	0.000*	1.8467	2.1210

Key: * indicates statistically significance at 5% level of significance, CI indicates credible interval, Sigma indicates variance and Ref is reference category

**Table 4:** Bivariate Bayesian linear mixed model result for hematocrit

Variables	Categories	Values	Standard error	*p*-values	95% CI	
					Lower	Upper
Intercept	-	46.6698	0.0995	0.000*	42.3961	50.6943
Visit time	-	-0.2834	0.0006	0.000*	-0.3193	-0.2473
WBC	-	-0.1166	0.0067	0.506	-0.4721	0.2380
RBC	-	1.5653	0.0115	0.035*	1.0089	2.1886
Platelet cell count	-	0.1088	0.0001	0.000*	0.0151	1.0028
Lymphocyte	-	-0.0076	0.0009	0.027*	-0.0114	-0.0078
Monocyte	-	-0.2168	0.0023	0.013*	-0.2489	-0.2032
Adherence (poor)	Fair	0.3929	0.0241	0.009*	0.0918	2.2026
	Good	1.7534	0.0299	0.026*	1.0676	3.6784
WHO(Ref = Stage-I)	Stage-II	-1.1901	0.0278	0.107	-1.7656	1.1438
	Stage-III	-0.3957	0.0299	0.008*	-0.7331	-0.0162
	Stage-IV	1.0194	0.0334	0.330	-1.0434	3.1248
ART regiment (Ref = 1d)	1c	-1.4337	0.0280	0.017*	-1.9249	-0.1126
	1e	-0.8277	0.0323	0.396	-2.7704	1.1987
	Other	-1.4003	0.0303	0.108	-1.8398	1.2178
OIs(Ref = No)	Yes	-1.7757	0.0305	0.044*	-3.7394	-0.0667
OCC(Ref = No)	Yes	0.6638	0.0340	0.572	-1.4207	2.8385
CD4 cell count (Ref = < 200cells/mm^3^)	>=200cells/mm^3^	0.2624	0.0212	0.692	-1.0501	1.5253
IPT(Ref = No)	Yes	0.5578	0.0244	0.043*	0.4897	2.0968
CPT(Ref = No)	Yes	0.1252	0.0318	0.045*	0.0898	2.1997
Viral load (Ref=>=10,000)	< 10,000	0.7071	0.0215	0.009*	0.0917	1.2351
Types of TB (Ref = pulmonary)	Extra-pulmonary	-0.2432	0.0361	0.792	-2.4326	2.1173
Sigma		2.3523	0.0030	0.000*	2.1969	2.5187

Key*statistically significance at 5% level of significance, CI indicates credible interval, Sigma indicates variance and Ref is reference category

## Discussions

In this retrospective cohort follow-up study the common clinical predictors that affected hemoglobin level and hematocrit jointly were red blood cell (RBC), platelet cell count, lymphocyte count, treatment adherence, WHO clinical stage, ART regiment, OIs, IPT drug users, and viral load count.

Patients RBC increased by one unit, the average hemoglobin level and hematocrit was increased by 0.1 g/dl($\beta =\text{0.1365,95}\% CI: (0.5477-1.2756$) and 1.6%($\beta =\text{1.5653,95}\% CI: (1.0089-2.1886$). These imply patients high RBC leads to high hemoglobin level and hematocrit. This leads to co-infected patients significantly associated with less mortality. The idea of theses result is consistent with previous study conducted in China [22]. However, the result of these findings is contradicted with previous study done in Pakistan’s [28].

Percentage lymphocyte count of patients increased by one unit, the average hemoglobin level and hematocrit was decreased by 0.02 g/dl ($\beta =-\text{0.0225,95}\% CI: -(0.0541-0.0116)$) and 0.01% ($\beta =-\text{0.0076,95}\% CI: -(0.0114-0.0078)$) respectively. These result is in line with former study done in China and Libya [22, 26]. World health organization clinical stage IV (β= -0.2695, 95% CI: -(1.4683 -0.0217)) and III(β=-0.3957, 95% CI: -(0.7331-0.0162)) are significant factors for hemoglobin level and hematocrit respectively. This indicated co-infected patients under the categories of WHO clinical stage-IV and III had 0.3 g/dl and 0.4% decrement in their average hemoglobin level and hematocrit than WHO clinical stage-I respectively. I.e. co-infected patients under this clinical stage had high progression of HIV results for low significant concentration of hemoglobin level and hematocrit.

Co-infected patients who treated 1e and other ART regiment had 0.2 g/dl (β = 1.2574, 95% CI: (0.2918–2.0930) and 1.04 g/dl (β = 1.0358, 95% CI: (1.0142–2.2088) increment in their average hemoglobin level than patents who treated 1d ART regiment. similarly, co-infected patients who treated 1c regiment had 1.4% (β=-1.4337, 95% CI: -(1.9249 − 0.1126)) decrement in their average hematocrit than patents who treated 1d ART regiment. The idea of this result is supported with previous study [23].

This study also showed that TB/HIV co-infected patients had 0.3 g/dl (β=-0.2734, 95% CI: -(-1.4240-0.0823)) decrement in their average hemoglobin level than patents with-out OIs. i.e. patents with OIs had affected by different AIDS related infections and results for a less average hemoglobin level than patents with-out OIs. This result is supported with a study done in Ethiopia [32]. Likewise, co-infected patients had 1.8% (β=-1.7757, 95% CI: - (3.7394 − 0.0667)) decrement in their average hematocrit than patents with-out OIs. The reduction of hematological parameter leads to high degree of mortality under OIs co-infected than with-out OIs. The idea of this result is supported with previous study [18].

TB/HIV co-infected patients under fair and good treatment adherence had 0.6 g/dl (β = 0.6118, 95% CI: ( 0.0740–1.4056) and 1.2 g/dl (β = 1.1726, 95% CI: ( 1.0160–2.2996) increment in their average hemoglobin level than poor treatment adherence. Similarly, TB/HIV co-infected patients under fair and good treatment adherence had 0.4% (β = 0.3929, 95% CI: ( 0.0918–2.2026) and 1.8% (β = 1.7534, 95% CI: ( 1.0676–3.6784) increment in their average hematocrit than poor treatment adherence. This implied co-infected patients who had poor treatment follow-up status leads to a reduction of hematological parameter. The idea of this result is supported with previous study [25].

IPT drug users co-infected patients had higher (β = 1.0404, 95% CI: (1.0052–2.9102) and (β = 0.5578, 95% CI: (0.4897–2.0968) average hemoglobin level and hematocrit than IPT non user patents. This indicated co-infected patients under IPT drug users had better survivalist than non-users. Likewise, patients’ whose viral load count < 10,000 copies/mL had higher ($\beta =0.5792, 95\% CI: (0.5208-2.2873$) and ($\beta =0.7071, 95\% CI: (0.0917-1.2351$) average hemoglobin level and hematocrit respectively than viral load count ≥ 10,000 copies/mL. This result indicated minimum viral load count had easily control virus concentration and leads to a higher hemoglobin level and hematocrit.

## Conclusion and recommandations

This study revealed that clinical predictors red blood cell count, platelet cell count, fair and good treatment adherence, other ART regiment, IPT drug users, and viral load count < 10,000 copies/mL, were associated with high hemoglobin level concentration while, lymphocyte count, WHO clinical stage-IV,1e ART regiment, and patients with OIs results for low hemoglobin level concentration. Likewise, red blood cell count, platelet cell count, fair and good treatment adherence, IPT drug users, and viral load count < 10,000 copies/mL co-infected patients had high hematocrit, while lymphocyte count, WHO clinical stage-III,1c ART regiment, and patients with OIs significantly leads to low hematocrit. FMOH or other Health professionals, program planers, decision makers, project implementers, government, and non-governmental organizations should be give special attention based on these important clinical predictors to minimize the risk of TB/HIV co-infected patients and improve their health status during the follow-up time. Health professionals give more attention to reduce progression of disease when the co-infected patients come back again in the hospital. In addition, the health staff should conduct health related education for individuals to examine continuous check-up of co-infected patients.

### Accronyms

ART: Antiretroviral therapy; BMI: Body Mass Index; CD4: Cluster Differentiation 4; CPT: Cotrimoxazole preventive therapy; DIC: Deviance Information Criterion; E.C: Ethiopian Calendar; FMOH: Federal Ministry of Health; HIV: Human Immune Deficiency Virus; IPT: Isoniazid preventive therapy; MRN: medical registration number; OCC: Other Comorbid Condition; OIs: Opportunistic Infections; PLWHIV: People Living With Human Immune Deficiency Virus; RBC: Red Blood Cell; TB: Tuberculosis; UNAIDS: The Joint United Nations Program on HIV/AIDS; WBC: White Blood Cell; WHO: World Health Organization.

## Data Availability

The data used in the current investigation is available from the corresponding author and can be attached upon request. The data accessed in the current investigation complied with relevant data protection and privacy regulations.
